# A Study of the Peripheral Vessels in Patients With Type 2 Diabetes Mellitus With or Without Foot Involvement

**DOI:** 10.7759/cureus.28542

**Published:** 2022-08-29

**Authors:** Pragateshnu Das, Debmalya Bhattacharya, Rajlaxmi Sathpathy

**Affiliations:** 1 Neurology, Kalinga Institute of Medical Sciences, Bhubaneshwar, IND; 2 Hematology, Apollo Hospital, Kolkata, IND

**Keywords:** peripheral arterial disease, ankle brachial pressure index, diabetic foot, diabetes mellitus, peripheral vascular disease

## Abstract

Peripheral vascular disease (PVD) is chronic limb ischemia caused by atherosclerosis of the peripheral arteries. Diabetes mellitus (DM) is a risk factor for this disease. The probability of a diabetic foot ulcer (DFU) is higher in a patient with DM and PVD than in a patient without DM. Ankle-brachial-pressure index (ABPI) allows the measurement of blood flow towards the distal extremities, which could help timely diagnosis, initiate brief therapy, and minimize the risk of critical limb ischemia and loss. This study aims to determine the prevalence of peripheral vascular disease and assess its association with intima-media thickness (IMT) in diabetic patients with and without foot ulcers in India. In the present study, we included all type 2 DM patients. The assessment was conducted clinically by measuring Ankle Brachial Pressure Index (ABPI) radiologically by Duplex Ultrasonography (Samsung HS 70A machine), and Doppler (Linear probe- LA3 - 12A) and IMT were detected. In healthy adults, IMT ranges from 0.25 to 1.5mm, and values above 1.0mm are often considered abnormal and linked with atherosclerosis and significantly increased cardiovascular disease (CVD). In this study, 72 patients with DM were enrolled over the study period; 52 patients presented with DFU, and 20 presented without DFU. The prevalence of PVD was higher in males compared to females; 40% of the patient population in the age range of 40-49 years was with PVD, and 62.5% of patients with PVD showed an IMT value more than 1.0mm, whereas only 5% patients without PVD shows IMT value more than 1.0mm. In conclusion, among type 2 diabetic subjects, the prevalence of PVD is 72.2%, and IMT is strongly associated with PVD.

## Introduction

Diabetes Mellitus (DM) is a chronic, metabolic disease characterized by increased levels of blood glucose, which result from absolute or relative insulin deficiency in circumstances with β-cell dysfunction, insulin resistance, or both [1]. It is one of the most usual and rapidly growing diseases worldwide. Diabetes is fast becoming a potential epidemic in India, with 77 million patients [2] and the vast majority still undiagnosed.

Diabetic foot is a condition in which foot ulcers form in patients with DM [3]. It is a frightening disorder with extended hospitalization and expensive with chances of an amputated extremity [4]. However, it is possible to prevent amputation using educational and care strategies [5]. Diabetic foot is characterized by a classical triad of neuropathy, ischemia, and infection [4]. The risk of a person with DM having a DFU has been reported to be as high as 25%. Diabetic foot is the most frequent cause (about 30%) of hospitalization in patients with DM [2]. 15-20% of patients with such DFU require an amputation. Nearly 85% of the amputations are preceded due to DFU. Several elements for developing DFU have been proposed, the significant being peripheral sensory neuropathy followed by peripheral vascular disease (PVD).

PVD is chronic limb ischemia which is always generated by atherosclerosis of the peripheral veins. PVD is one of Type II DM's most common macrovascular complications [6]. Prevalence increases with age, about 3% in people below 60 years of age, and rises to over 20% in people over 75. Only a quarter of people with PVD are symptomatic. Apart from age, other risk factors include smoking, diabetes mellitus, hypertension, physical inactivity, and obesity. PVD is a disease affecting the veins supplying the legs, feet, kidneys, and intestines. PVD is typically more severe in patients with diabetes than in comparable nondiabetic individuals and is associated with a worse prognosis [7,8]. Patients with PVD risk myocardial infarction, stroke, and mortality [9,10]. Early detection of vascular changes helps effectively handle DM and DM complications.

The diagnosis technique further affects the magnitude of PVD [7]. A more precise assessment of PVD in DM must depend on a validated and reproducible diagnosis technique. Such a test includes the ankle-brachial index (ABI). The ABPI is the ratio of the ankle to systolic brachial pressure. It is suggested to be calculated by dividing the higher systolic pressure of the dorsalis pedis and tibialis posterior vessels at the ankle by the higher systolic pressures measured in the brachial vein [11]. ABPI is a simple and noninvasive technique. The ABPI showed to be more accurate and verified against angiographically confirmed disease and found to be 95% sensitive and nearly 100% specific [12].

There is a need for a structured evaluation of PVD in all DM patients. The information can help prepare protocols for the effective management of DM patients to limit morbidity. The present study aims to determine the prevalence of peripheral vascular disease and assess its association with ankle-brachial index in diabetic patients with and without foot ulcers in India.

## Materials and methods

A retrospective study was carried out on 72 consecutive patients with type 2 DM attending the medicine outpatient department/ward. Endocrine outpatient department/ward and diabetic foot clinic of SCB Medical College and Hospital, Bhubhaneshwar, between 2011 to 2012, as the patients were monitored for changes observed under the same categories for 10 years. All patients with type 2 diabetes mellitus were included in the study, and the patients with trauma, Buerger disease, fibromuscular dysplasia, or vasculitis were excluded. Ethical approval was obtained from the SCB Medical College Ethics Committee (1536/IEC/ SCBMCH/2022).

A detailed general, systemic, and foot examination of patients was done. The body mass index (BMI) was calculated using the formula: weight (kg)/height (m2). A fasting blood sugar (FBS), postprandial blood sugar (PPBS), hemoglobin [R1] (HB), cholesterol, high-density lipoprotein (HDL), low-density lipoprotein (LDL), very low-density lipoprotein (VLDL) and IMT were measured. Intima medial thickness of the popliteal vein is predictive of peripheral vascular disease, which can be detected using ABPI. ABPI was measured radiologically by Duplex Ultrasonography using an ACUSON 128 × P/10 machine with a 7.5 MHz linear superficial array probe in B- mode. B-scan was used for detecting IMT, pulse wave signal for flow velocity, and color flow for site determination. 

The statistical analysis of all mentioned clinical parameters was performed. The continuous variables are expressed as mean ± standard deviation (SD). A comparison of variables was performed using ANOVA tests. Variables with a P-value < 0.05 was considered as the level of significance.

## Results

Table 1 shows the demographic factors of patients (n=72) enrolled in this study. Of the total patients, it was found that 73.1% are males and 26.9% are females with PVD, whereas 60% are males and 40% are females without PVD. Age distribution was shown in table 1, and here we found 40.4% of patients falling in the 40-49 years of age group are found with PVD, and 45% of patients in the age group of 50-59 years are found without PVD. In the case of the distribution of BMI, 73.1% are in the range of 18.5- 22.9 with PVD, and 70% are in the range of 18.5- 22.9 without PVD.

**Table 1:** Demographic factors in patients with PVD and without PVD PVD: Peripheral vascular disease

Demographic factor		With PVD		Without PVD	
		Frequency	Percent	Frequency	Percent
Gender	Male	38	73.1	12	60.0
	Female	14	26.9	8	40.0
	Total	52	100.0	20	100.0
Age (years)	30-39	1	1.9	1	5.0
	40-49	21	40.4	4	20.0
	50-59	16	30.8	9	45.0
	60-69	10	19.2	5	25.0
	70-79	4	7.7	0	0
	80-89	0	0	1	5.0
	Total	52	100.0	20	100.0
BMI (kg/m^2^)	18.5 – 22.9	38	73.1	14	70.0
	23 – 24.9	10	19.2	4	20.0
	25 – 29.9	4	7.7	2	10.0
	Total	52	100.0	20	100.0

**Figure 1:**
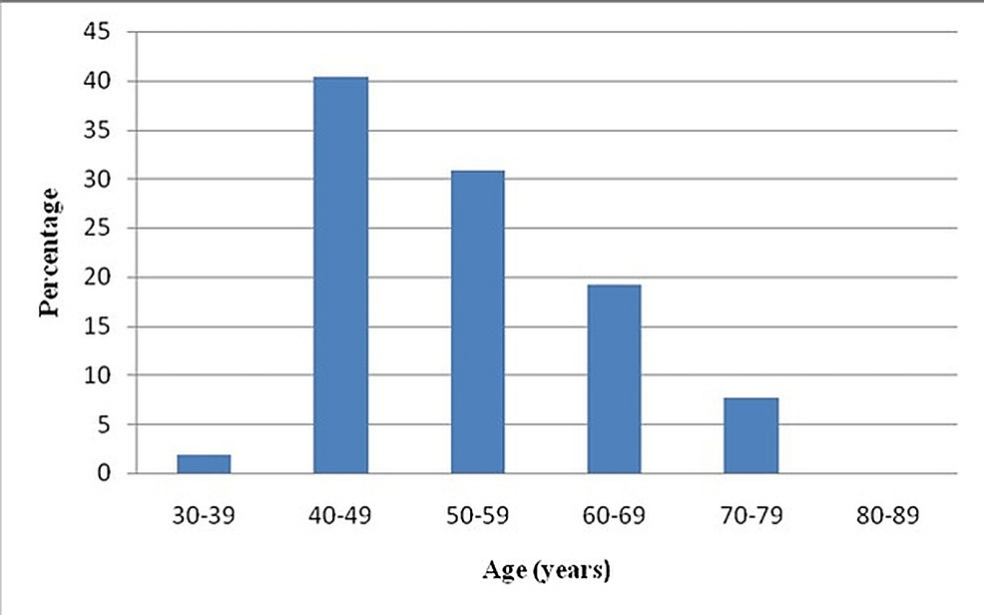
Graphical representation of Age-wise distribution of patients with PVD *x-axis: age (years), y-axis: percentage (%) PVD: Peripheral vascular disease

**Figure 2:**
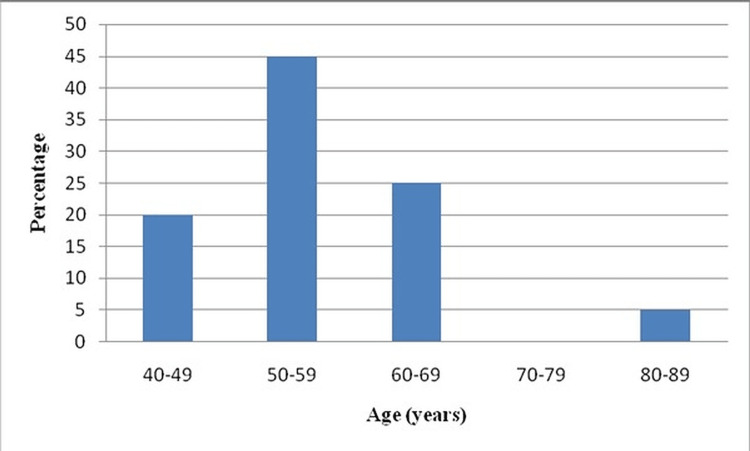
Graphical representation of Age-wise distribution of patients without PVD *x-axis: age (years), y-axis: percentage (%) PVD: Peripheral vascular disease

Out of the total patient population, 40% was in the age range of 40-49 years for patients with PVD, whereas 45% was in the age range of 50-59 years for patients without PVD. A graphical representation of the age distribution is shown in Figures 1, 2.

The result of statistical analysis data, i.e., mean, standard deviation, and range for demographic, clinical, and biochemical factors for patients (n= 52) with PVD, are shown in table 2.

**Table 2:** Statistical analysis for patients with PVD PVD: Peripheral vascular disease, FBS: fasting blood sugar; PPDS: postprandial blood sugar; HB: hemoglobin; HDL: high-density lipoprotein; LDL: low-density lipoprotein; VLDL: very low-density lipoprotein; IMT: intima-media thickness; RT: right; LT: left.

Factors	Mean	Standard deviation	Range
Age (years)	54.48	9.03	42.00-76.00
FBS (mmol/L)	172.42	42.35	92.00-380.00
PPBS (mmol/L)	208.56	47.99	147.00-440.00
HBA1C (%)	9.14	1.81	6.50-14.70
CHOLESTEROL (mg/dL)	194.17	27.28	110.00-273.00
TRIGLYCERIDE (mg/dL)	204.83	68.83	138.00-544.00
HDL (mg/dL)	40.38	14.05	28-58
LDL (mg/dL)	111.27	33.63	64-171.00
VLDL(mg/dL)	28.06	12.67	14-60.00
IMT-RT (mm)	1.13	0.19	.69-1.50
IMT-LT (mm)	1.09	0.19	.74-1.40
BMI (kg/m^2^)	22.22	1.77	19.60-28.8

The result of statistical analysis data, i.e., mean, standard deviation, and range for demographic, clinical, and biochemical factors for patients (n= 20) without PVD, is shown in table 3.

**Table 3:** Statistical analysis for patients without PVD PVD: Peripheral vascular disease, FBS: fasting blood sugar; PPDS: postprandial blood sugar; HB: hemoglobin; HDL: high-density lipoprotein; LDL: low-density lipoprotein; VLDL: very low-density lipoprotein; IMT: intima-media thickness; RT: right; LT: left.

Factors	Mean	Standard deviation	Range
Age (years)	55.05	9.83	33.00-80.00
FBS (mmol/L)	161.30	35.32	96.00-220.00
PPBS (mmol/L)	206.55	40.80	147.00-305.00
HBA1C (%)	7.46	0.60	6.50-8.90
CHOLESTEROL (mg/dL)	173.70	25.30	95.00-220.00
TRIGLYCERIDE (mg/dL)	192.95	57.19	120.00-329.00
HDL (mg/dL)	39.95	6.31	30.00-50.00
LDL (mg/dL)	101.50	18.34	55.00-147.00
VLDL(mg/dL)	19.10	4.98	10.00-30.00
IMT-RT (mm)	0.70	0.18	.41-1.30
IMT-LT (mm)	0.72	0.17	.39-1.23
BMI (kg/m^2^)	22.46	1.99	19.80-27.6

The statistical comparison of clinical and biochemical characteristics of the study groups with PVD (n = 52) and without PVD (n =20) are shown in Table 4.

**Table 4:** The statistical comparison of data of patients with PVD and without PVD PVD: Peripheral vascular disease, FBS: fasting blood sugar; PPDS: postprandial blood sugar; HB: hemoglobin; HDL: high-density lipoprotein; LDL: low-density lipoprotein; VLDL: very low-density lipoprotein; IMT: intima-media thickness; RT: right; LT: left.

	WITH PVD	WITHOUT PVD	p-value
FBS (mmol/L)	172.42±42.34	161.30±35.31	0.30
PPBS (mmol/L)	208.56±47.98	206.55±40.80	0.86
HBA1C (%)	9.14±1.81	7.46±0.59	0.0001
TRIGLYCERIDE (mg/dL)	204.83±68.83	192.95±57.19	0.49
CHOLESTEROL (mg/dL)	194.17±27.28	173.70±25.30	0.004
HDL (mg/dL)	40.38±14.05	39.95±6.31	0.894
LDL (mg/dL)	111.27±33.63	101.50±18.33	0.224
VLDL(mg/dL)	28.06±.12.67	19.10±4.98	0.0031
IMT-RT (mm)	1.12±.19	0.70±0.18	0.001
IMT-LT (mm)	1.09±.185	0.71±0.17	0.001
BMI (kg/m^2^)	22.22±1.76	22.46±1.99	0.624

As per table 4, the mean IMT in subjects with PVD (n = 52) was 1.12 ± .19mm (right) and 1.09 ± .18mm (left) whereas in subjects without PVD (n = 20), it 0.70 ± 0.17mm (right) and 0.71 ± 0.17mm (left). Values of HBA1c (p-value = 0.0001), Cholesterol (p value= 0.004), VLDL (p value= 0.0031) and IMT (p value=0.001) show significance. The distribution of IMT results in patients with PVD and without PVD is shown in table 5.

**Table 5:** The distribution of IMT results in patients with PVD and without PVD PVD: Peripheral vascular disease, IMT: intima-media thickness

WITH PVD			WITHOUT PVD		
IMT (mm)	FREQUENCY	PERCENT	IMT (mm)	FREQUENCY	PERCENT
<0.7	1	1.9	<0.7	7	35.0
0.7-1.0	19	36.5	0.7-1.0	12	60.0
>1.0	32	61.5	>1.0	1	5.0
TOTAL	52	100.0	TOTAL	20	100.0

## Discussion

The true magnitude of PVD in DM patients is challenging to measure as many patients can be asymptomatic, and some do not report their symptoms. ABPI is an excellent initial screening tool for the assessment of PVD. This study aimed to determine the prevalence of peripheral vascular disease and assess its association with ankle-brachial index in diabetic patients with and without foot ulcers in India. It was reported that DM patients with evidence of systemic atherosclerosis were found to be at risk for PVD. The prevalence of diabetes mellitus in India is 8.8% (among people between 20-79 years of age). The actual prevalence of PVD in people with DM is challenging to calculate, as most cases are asymptomatic and many cases refrain from reporting the symptoms. DFUs were found in 4.54% of patients newly diagnosed with type 2 diabetes mellitus in India [2]. Earlier estimations for the magnitude of PVD among DM subjects in the United States and Europe vary from 9.5-42%. In contrast, for the Asian region, the magnitude of DM populations has been reported to be lower than that in the western community [10].

In the present study, 72 persons with diabetes were enrolled over the study period; 52 patients presented with DFUs, and 20 patients were without DFUs. In this study, the prevalence of PVD in DM subjects was found to be 72.22%, with 52 out of 72 patients showing the presence of PVD. As reported earlier by Pradeepa R et al. [10], the prevalence of PVD is higher in DM subjects than in non-DM subjects in population-based and clinic-based studies.

The prevalence of PVD, symptomatic and asymptomatic, is higher in males than in females in this study. It was found in this study that gender-wise distribution indicates 73% of males and 27% of females with PVD, whereas 60% was male and 40% was female without PVD. Thus the magnitude of PVD in DM patients was higher in males than in females. These results contrast with Pradeepa R et al. [10], where female subjects were more likely to have PVD than male subjects.

The age-wise distribution shows that 40% of the patient population was in the age range of 40-49 years for patients with PVD, whereas 45% was in the age range of 50-59 years for patients without PVD. Like Ikem R et al. [7], age showed statistical significance in PVD risk in the present study.

The ABPI assessment was done radiologically by Duplex Ultrasonography which determines IMT. The normal IMT value ranges from 0.25 to 1.5mm in healthy adults, and values above 1.0mm are often considered abnormal [13] and are linked with atherosclerosis and increased cardiovascular disease. In present study, 62.5% patients with PVD shows IMT value more than 1.0mm, 36.5% patients shows IMT value in between 0.7- 1.0mm and 1.9% patients show IMT value below 0.7mm. In this study, 5% of patients without PVD showed an IMT value of more than 1.0mm, 60% of patients showed an IMT value between 0.7- 1.0mm, and 35% of patients showed an IMT value below 0.7mm. That means IMT is strongly associated with PVD.

The PVD prevalence is symptomatic and asymptomatic, and it is more in males than in females in this study. It is thus essential to investigate the presence of PVD in asymptomatic cases to manage the risk elements as early as possible and minimize mortality. We recommend screening for PVD while on the time of detection of DM for both timely detection and to prevent the disease.

## Conclusions

This study concluded that type 2 diabetes patients with IMT of more than 1.0mm, increased CVD, and diabetic foot ulcers were prone to more risks of peripheral vascular disease. It also revealed that these risks increased gradually with the increase of age. This study revealed that males tend to have more risk of PVD than females. Detection of peripheral vascular disease in diabetic foot patients using Duplex Ultrasonography and ABPI together with regular clinical analysis will help to assist timely detection of critical extremities. The patients may not all be symptomatic or show clear signs of PVD though they need to be examined for the presence of PVD. The current and earlier studies have repeatedly manifested the need and advantages of investigating DM for peripheral ischemia to give better care. The care of DM subjects must be initiated with preventive actions, which is the key to avoiding further complications.
